# In search of sex-related mediators of affective illness

**DOI:** 10.1186/s13293-021-00400-4

**Published:** 2021-10-18

**Authors:** Christopher Sikes-Keilp, David R. Rubinow

**Affiliations:** Department of Psychiatry, University of North Carolina, Chapel Hill, NC USA

**Keywords:** Sex differences, Depression, Reproductive steroids, Brain, Stress, Network connectivity, Neurotransmitter, Cell signaling, Epigenesis

## Abstract

Sex differences in the rates of affective disorders have been recognized for decades. Studies of physiologic sex-related differences in animals and humans, however, have generally yielded little in terms of explaining these differences. Furthermore, the significance of these findings is difficult to interpret given the dynamic, integrative, and highly context-dependent nature of human physiology. In this article, we provide an overview of the current literature on sex differences as they relate to mood disorders, organizing existing findings into five levels at which sex differences conceivably influence physiology relevant to affective states. These levels include the following: brain structure, network connectivity, signal transduction, transcription/translation, and epigenesis. We then evaluate the importance and limitations of this body of work, as well as offer perspectives on the future of research into sex differences. In creating this overview, we attempt to bring perspective to a body of research that is complex, poorly synthesized, and far from complete, as well as provide a theoretical framework for thinking about the role that sex differences ultimately play in affective regulation. Despite the overall gaps regarding both the underlying pathogenesis of affective illness and the role of sex-related factors in the development of affective disorders, it is evident that sex should be considered as an important contributor to alterations in neural function giving rise to susceptibility to and expression of depression.

## Introduction

Sex is increasingly regarded as an important contributor to the development of mental illness, including affective disorders, neurodevelopmental disorders, and addiction. Since 2014, it has been NIH policy that grant applications must address the possible role of sex in the proposed study. With regards to affective disorders, sex differences in the prevalence and symptomatology of major depressive disorder (MDD), anxiety, and post-traumatic stress disorder (PTSD) have been known for decades, with females experiencing these disorders at approximately twice the rate as males [1]. In general, studies of sex-related differences in animals and humans have focused on specific, often single, measures that have yielded little in terms of explaining the overall sex differences in depression evident in epidemiological studies. Our current lack of understanding is not surprising, given the complexity of the relationship between sex and brain function, as well as the confusing array of evidence regarding the biological underpinnings of affective illness. The impact of sex widely ranges from direct influence on central nervous system processes (through genetic sex and gonadal steroids) to indirect effects, such as those elicited from the environment (e.g., as a result of social and cultural expectations). From a mechanistic perspective, sex encompasses enduring effects of exposure to sex hormones during critical developmental periods (organizational effects), transient effects of sex hormones (activational effects), interactions between organizational and activational effects, and genetic effects (i.e., having two X chromosomes vs. one Y). Mapping sex onto the processes underlying affective regulation, which are similarly complex and dependent on contextual factors (such as timing during the lifespan, prior experience, genetic background, species, and stimulus, all of which interact with sex), may, therefore, be best served by the organization of existing knowledge into a framework that allows us to think more broadly about the role of sex in dynamic emotional states. In this overview, we attempt to create such a framework, illustrating with examples the broad conceptual levels at which sex differences have been described in the brain and conceivably could contribute to female predominance of affective disorders. Our current knowledge can be roughly divided into several categories, with sex effects having been identified in brain structure, brain connectivity, signal transduction, transcription/translation, and epigenesis. Each of these categories will be discussed below with examples of key findings of sex differences related to affective function, though a fully comprehensive review is clearly precluded by the scope of the topic. In our discussion, we reflect on the importance and limitations of this work, as well as offer perspectives on the future of research on sex differences as they relate to affective disorders.

Though “affective disorders” encompass a broad range of illnesses, including MDD, bipolar disorder, anxiety disorders, and PTSD, this review primarily utilizes examples from depression and depressive-like behavior for the following reasons: (1) an abundance of animal models exist for depressive-like behavior [2]; (2) sex differences in prevalence are well-established [3]; (3) reproductive events during the lifespan (e.g., pregnancy, menopause) alter the expression and substrates of depression [4, 5]; (4) a comparatively large body of preclinical and clinical research exists pertaining to sex differences in depression; and (5) depression is either a core feature of or highly comorbid with the other disorders listed [6, 7].

In addition, we have chosen to view the relationship between sex and affect regulation through the lens of stress and the stress response for several reasons. First, stress is a critical factor in both the precipitation of and susceptibility to affective disorders, demonstrated in numerous epidemiologic studies (e.g., the Adverse Childhood Experiences study, which found relationships between early stress and depression, substance use, and suicide [8–10]). Abundant evidence also confirms early observations of depression-related disturbances in the stress axis (including cortisol hypersecretion, impaired negative feedback, and corticotropin-releasing hormone (CRH) dysregulation) [11–15]. Second, there is a rich literature describing sex differences in the stress response, both in animals and humans [16–22]. Third, stressors in animal studies are the means of inducing behaviors believed to model symptoms of affective disorders in humans, thus permitting investigation of potential biological mediators of these disorders and associated sex differences. It should be noted that context-determining factors interacting with the stress response, such as developmental stage/aging, prior experience (e.g., adversity), and sex-specific life events (e.g., pregnancy, menopause), are known to dramatically alter both physiologic and behavioral responses and may be required for expression of sexually dimorphic traits [4, 23–25]. Although certain examples are highlighted below, a full discussion of the critical role of context in the origin and expression of sex differences in affective dysfunction is beyond the scope of this review.

Finally, as noted above, when considering the evidence for a role of sex in the biology of affect regulation, one must keep in mind the multiple means by which sex can influence biology (e.g., is the difference hormone-dependent or not). The impact of sex chromosomes independent of hormones is not well-understood, in large part due to the difficulty in distinguishing the effects of genetic sex from those of gonadal sex [26, 27]. However, several lines of emerging research are beginning to elucidate a substantial role for genetic complement on sexual differentiation and function. Higher rates of certain neurodevelopmental and affective disorders have been observed in sex chromosome aneuploidies (in which sex chromosomes are present in abnormal quantities) [28], and heritability analyses have suggested a significant influence of sex-chromosomes on brain anatomy [29]. In addition, the four-core genotypes (FCG) model (described in detail in a later section) is a preclinical paradigm that makes possible the separation of gonadal sex and genetic sex, allowing for direct comparison of different gene complement/hormone profile combinations [26]. Within the category of hormone-dependent effects, differences may arise as a result of organizational/programming effects, acute/activational effects, or a combination of the two. Organizational effects occur consequent to exposure to sex-steroids during critical periods of development and persist irrespective of subsequent changes in hormone levels. Important demonstrations of these programming effects are illustrated in the classic studies of Phoenix et al., Gorski et al., and Arnold et al., which established that behavioral capacities in adulthood (e.g., aggression, sex behaviors) are dependent upon these perinatal exposures [30–32]. Organizational effects, in addition to associated effects on brain morphology [33, 34], are a product of sex-steroid regulation of many of the fundamental processes of brain development, including neuroplasticity, epigenesis, and immunoregulation [35–37]. Activational effects, on the other hand, comprise the immediate and reversible effects of sex hormones and are mediated largely, albeit not exclusively, through sex hormone receptors. Sex hormone receptors are ubiquitous in the central nervous system, and there is virtually no element of neural function that is not regulated by sex hormones. Sex steroids can acutely regulate neural structure, excitability, cell function, and transmission [38], effects which ultimately extend to the level of brain circuits and global brain function. At the interface of organizational and activational effects are those that cannot occur without both early exposure to and current presence of a hormone, i.e., an acute effect programmed by a developmental one. For instance, male rats castrated at birth show incomplete mating behavior upon re-exposure to testosterone in adulthood, a pattern that is not seen in males castrated in adulthood [39, 40]. The impact of organizational and activational effects and their interactions must be disentangled to understand how observed sex differences are produced. Methods employed (such as four-core genotypes) often reflect the effort to decompose the underlying mechanisms of hypothesized sex differences. Complexity of effects and mechanisms notwithstanding, the implications from the findings summarized below suggest that sex is a powerful acute and developmental context and must be considered as a critical potential contributor to alterations in neural function giving rise to susceptibility to and expression of depression.

## Brain structure

Structural brain differences between males and females of various species have been described since the latter half of the twentieth century, with evidence for sex differences firmly established by the seminal discovery of sexually dimorphic brain regions responsible for vocal control in songbirds [41]. In humans, women have been observed to have a higher percentage of gray matter volume relative to white matter [42, 43], as well as greater volumes of the orbital frontal cortices [44]; men appear to have higher gray matter densities in several brain regions, including amygdala, hippocampus, insular cortex, and putamen [45]. Developmentally, men appear to obtain peak brain volumes at a later age than women, and characteristics such as brain volume, directional organization, and myelination of many regions have been shown to vary by sex in adolescents [46, 47]. In this section, we first explore how differences in affect-regulating brain areas may predispose female animals to an increased CNS response to stress. We then review clinical findings, with a focus on structural brain differences between males and females exposed to childhood trauma.

### Preclinical findings

While structural changes in the brains of depressed individuals have been observed independent of sex [48–50], there is little conclusive evidence relating structural differences to sex differences in depression. Preclinical findings, however, suggest that regions implicated in affective processing, such as the locus coeruleus, contain sexually dimorphic features that may play a role in increased female vulnerability to depressed states. The locus coeruleus (LC) directs attention and mediates arousal via the integration and relay of stress signals to and from the HPA axis. While important for adaptive responses to environmental stimuli that threaten survival, the LC-norepinephrine system demonstrates amplified reactivity following chronic stress, resulting in pathological behaviors resembling anxiety in animal models [51]. Unsurprisingly, it has been hypothesized that dysfunction of this system underlies hyperarousal states characterizing human anxiety and trauma related disorders [52, 53]. In female rats, the LC dendritic processes synapsing on terminals (originating in the amygdala) that release corticotropin releasing factor (which both activates the HPA axis and acts as a central neuromodulator) demonstrate longer trees, with more branches and longer branch lengths, and have a greater number of synaptic contacts [54]. This increased complexity ultimately results in a framework for more emotion-related information to be transmitted by the amygdala in response to stress in females, and represents a potential link between structure, HPA-axis/arousal response, and vulnerability to affective illness. It is also important to note that the effects of stress on regional morphology in various brain regions, particularly following prenatal stress, have been shown to vary by sex in animal models [55–60].

### Clinical findings

Previous studies of brain structure in major depressive disorder were mostly underpowered to detect sex differences (in those that included both men and women), and single-sex studies offer little in the way of comparative data [61, 62]. Further hampering comparisons, it is likely that individual men and women represent mosaics, where each individual brain is a composite of male-typical and female-typical features [63]. Indeed, recent MRI findings support both the extensive overlap between individuals and the emergence of sex differences only at a group level [63]. Nonetheless, recent evidence suggests that brain structure within regions implicated in depression is affected in a sexually dimorphic fashion by prenatal and childhood stress, representing a possible structural link between early exposure and subsequent susceptibility. A meta-analysis of data obtained by the Enhancing Neuroimaging Genetics through Meta-Analysis (ENIGMA) consortium demonstrated that, in cases of childhood maltreatment, greater maltreatment severity was associated with lower gray matter thickness and caudate volumes in adolescent and adult females, whereas in males, greater maltreatment severity was associated with decreased thickness of rostral anterior cingulate cortex [64]. Postnatal maternal depression has been associated with greater fractional anisotropy of the amygdala in female children [65], and several studies have demonstrated sex-specific effects of prenatal maternal stress on subsequent amygdala structure in newborns, with differential effects seen on volume [65–68] and microstructure [69]. One recent study demonstrated sex-specific effects of early perinatal stress on cortical gyrification, with young adult women who were previously exposed to stress either in-utero or during the first 18 months of life showing higher temporal gyrification and greater propensity for mood disturbance [70].

### Summary

Despite the limitations described above, as well as our poor understanding of the relationship of brain structure to depression generally (and potentially limited contribution of structure to depression overall), sex differences in specific brain regions implicated in affective function provide plausible explanations for findings of differential stress processing and susceptibility to depression. For example, sex differences in brain function under stressful conditions (e.g., learning is facilitated by stress in male rodents under certain conditions and impaired in females [71]) not only represent differential activation of certain regions and circuits, but as well are associated with dimorphic microstructural differences, such as synapse concentration [71]. Similar sex-dependent morphological differences have been identified in rodent mPFC pyramidal neurons [72] following repeated stress, a model for depression. It is, therefore, conceivable that regional sex-differences in brain structure—either innate or acquired—may contribute to the well-studied effects of sex-steroids on emotion processing [73] in influencing sex-dependent susceptibility to disturbances in affective regulation.

## Network connectivity

There is extensive evidence for effects of sex and sex steroids on neural processes related to network development and function [38, 74–77]. First, sex differences have been described in networks subserving emotional valence [78], pain and pain sensitivity [79], resting state function of the amygdala during adolescence [80] and in autism [81], and neurocognitive function [82]. Diffusion tensor imaging (DTI) studies have demonstrated higher fractional anisotropy and lower mean diffusivity of major white matter tracts in men [83–85], while studies of functional cortical connections suggest that female connectivity patterns are characterized by less laterality [86] but greater local and global connectivity [87, 88]. While the implications of these findings may be unclear, Ingalhalikar et al. postulated that, based on their analysis of the “structural connectome,” male brains are optimized for perception and coordinated action through *intra*hemispheric communication, while female brains are more adept at relaying information between analytical and intuitive processing modes via *inter*hemispheric communication [89] (although it should be noted that the authors’ conclusions have raised many questions—see [90]). In addition, sex hormones are known to exert a substantial influence on network function. PET and fMRI studies in humans have shown neuroregulatory effects of estradiol on working memory [91–93], reward [94–97], default mode function [98–100], emotional processing [73, 101–104], and components of the salience network [102, 105, 106], and functional connectivity effects have more recently been demonstrated for progesterone [107], particularly with regards to network changes across the menstrual cycle [108, 109]. Comparative data (between males and females) for hormonal effects on network function is relatively limited, though one recent study noted a potentially protective effect of endogenous estradiol against the deleterious effects of visceral adipose tissue on network covariance associated with cognitive decline in aging women, but not men [110].

Depression in both sexes has been characterized by alterations in the activity and connectivity of multiple, relevant CNS networks. Increases in default mode network (DMN) activity and decreases of the salience and central executive networks have been observed, which have been suggested as physiologic substrates of the increased rumination and decreased responsiveness to external stimuli often seen in depressed states [111, 112]. Aberrations in reward circuitry have been repeatedly documented [113], bearing a plausible association to the cardinal depression symptom, anhedonia. Changes in blood flow to and from critical nodes within the corticolimbic system, such as prefrontal cortex and amygdala, have also been shown to be altered in major depression [114, 115] and following stress in animal models [116]. Below, we present preclinical and clinical evidence for sex differences in network function in stress/affective disorders. Clinical research findings suggest a particularly important role of pubertal maturation in the development of network sex differences, consistent with the aforementioned effects of sex steroids [117].

### Preclinical findings

The existing preclinical evidence supports the idea that sex influences how antecedent stress shapes early organization and mature function of such networks. For instance, several studies have documented sexually dimorphic changes in functional connectivity following repeated stress between brain regions associated with the default mode network [118, 119] (e.g., between hippocampus and amygdala [119] and between prefrontal cortex and amygdala [118]). Differential circuit activation by exogenous administration of corticotropin-releasing factor has been observed in adult rats [120], suggesting one possible mechanism underlying this dimorphism. Differences in network function may also be reflected in sex-specific microstructural changes (i.e., at the neuronal level) that underlie network organization. Dendritic remodeling has been shown to occur in adult male rats, but not females, in hippocampal CA3 neurons following chronic restraint stress [121]. Adult female, but not male, rats show hormone-dependent selective dendritic remodeling in mPFC neurons projecting to basolateral amygdala in response to stress (males instead show remodeling of mPFC neurons projecting elsewhere) [122, 123]. Ovariectomy abolishes these mPFC changes, and estradiol addback to gonadectomized females increases mPFC dendritic branching, irrespective of the downstream target [122]. One study in adult rats demonstrated sex differences in several aspects of function in basolateral amygdala, including increased neuronal firing rates, more dendritic spines, and greater sensitivity/responsivity to glutamate in females [124]. Investigators noted that estrous cycle shifts in neuronal activity paralleled the rate of cued fear extinction, suggesting that activational hormonal effects produce identifiable CNS changes related to subsequent behavioral outcomes [124]. Sex-specific effects of stress on non-neuronal cell populations that affect neural circuitry, such as microglia, have also been observed [125].

### Clinical findings

While there is, on the whole, a paucity of research reporting human sex differences in functional connectivity related to depression, recent evidence from specific subpopulations has suggested that depressive and anxious symptomatology may have different network correlates in men and women. Higher “internalizing” symptoms (which correlate with depressive/anxious symptomatology) in female, but not male, adolescents have been associated with greater resting-state connectivity between amygdala and regions implicated in emotional and somatosensory processing, salience detection, and action selection, including cingulate gyrus, insula, and somatosensory cortices [126]. Similarly, intrinsic functional connectivity (iFC) of the DMN appears to weaken with pubertal maturation in females (compared to strengthening in males), with decreased iFC of the anterior cingulate within the DMN predicting higher internalizing symptoms later in adolescence [127]. Connectivity may vary as a function of sex and diagnostic classification (e.g., MDD vs. control) as well, with one study of adolescents demonstrating increased connectivity strength between cerebellum and superior frontal gyrus with age in male controls, but decreased connectivity with age in males with MDD (no effects were seen in females) [128]. Major depression in adult chronic ketamine users has been shown to have sex-specific resting-state connectivity patterns, with women showing increased connectivity between subgenual anterior cingulate cortex (sgACC) and dorsomedial prefrontal cortex and men showing increased connectivity between sgACC and bilateral superior temporal gyrus [129].

Even when sex itself is not a variable, studies of the effects of hormone fluctuations, either naturally occurring or induced, are consistent with the notion that sex steroids modulate brain dynamics relevant to mood. For one, manipulations of estradiol and progesterone have been shown to induce depressed states in certain women [130–132]. In addition, in studies of premenstrual dysphoric disorder (PMDD), the luteal (symptomatic) phase is associated with differential task-related activation of affect-relevant brain regions (e.g., amygdala, dorsolateral prefrontal cortex, medial prefrontal cortex, insula, orbitofrontal cortex) in adult patients compared with non-PMDD controls [73, 106, 133–136]. Manipulations employing GnRH agonists (which lead to suppression of ovarian hormone release through pituitary desensitization) produce reductions in PMDD symptoms with GnRH treatment [132, 137], with subsequent addback of estrogen and progesterone producing not only recurrence of symptoms but alterations in neural activity in regions and networks subserving mood [138, 139] and cognition [93]. As an example, one recent study found decreased resting regional blood flow in subgenual cingulate, a hub for affect regulation, following both estrogen and progesterone addback after leuprolide treatment in adult PMDD participants, but not controls [138].

### Summary

Given that brain regions implicated in affective disorders, such as hippocampus, amygdala, hypothalamus, and brainstem are rich in steroid hormone receptors [140], and that sex- and sex hormones exert a substantial influence on both mood and processes associated with network function, it is reasonable to infer a significant role of sex on network function related to affect. While affective disorders secondary to reproductive endocrine changes, such as postpartum depression, PMDD, and perimenopausal depression, are the most obvious examples of sex affecting neurocircuitry underlying behavioral state kinetics, reported sex differences in network function in depressed/stressed states suggest that meaningful sex effects exist beyond those generated by hormones. Sex as a whole is multifaceted and more complex than the acute effects produced by changes in hormone levels, encompassing genetic complement and organizational effects (and the interactions between them all) as well. Sex is also only one determining factor in the expression of network states, interacting with individual trait characteristics (e.g., genetic factors) to influence brain connectivity [138]. Nevertheless, the studies described clearly suggest that certain features of sex can not only be isolated and examined for their relationship to affect and neural function, but as well are likely to yield specific behavioral and neural findings that deepen our understanding of the pathogenesis of depressed states.

## Signal transduction

Sex hormones affect many, if not all, neurotransmitter systems in myriad ways [140]. Both excitatory and inhibitory effects of estradiol on several neurotransmitters, including glutamatergic [141–144], GABAergic [145, 146], dopaminergic [147–152], serotonergic [153–157], and noradrenergic [158–160], have been extensively documented. These effects occur via multiple mechanisms (including synthesis [153], release [149], turnover/degradation [161], receptor trafficking [154], and transport [162]) and are dependent on contextual factors, such as receptor subtype [163], brain area [164], developmental stage [165], duration of treatment/time following exposure [166, 167], mode of administration [167], and amount of steroid present [168]. Findings from both human and animal research suggest that these hormone–neurotransmitter interactions have meaningful functional consequences. For example, in animal studies, estradiol interacts with dopamine to influence reward decision-making and memory, with high estradiol states generating bias toward smaller, more accessible rewards [169] and preferential use of certain memory strategies [170] (This relationship appears to be modulated by individual baseline dopamine processing, with evidence in humans that working memory performance following estradiol exposure is either enhanced or impaired depending on genetic background [171]). Progesterone has a similarly complex relationship with various neurotransmitter systems, with effects distinct from (and at times opposite to) estradiol [140, 168]. Of considerable interest, allopregnanolone, a neurosteroid metabolite of progesterone, is a positive allosteric modulator of GABA-A receptors and facilitator of GABA’s inhibitory action, which has been implicated in the etiology of several affective disorders, including postpartum depression (PPD) [172, 173]. The precipitous decline in progesterone/allopregnanolone levels and resultant decrease in GABAergic transmission following delivery is hypothesized to underlie decreased mood and increased anxiety experienced by women susceptible to PPD [174], a suggestion supported by the recent approval of Brexanolone, a synthetic version of allopregnanolone, for the treatment of PPD [175, 176]. The allopregnanolone withdrawal hypothesis, however,  would not explain the development of depression during pregnancy. Nonetheless, the relevance to affective regulation of allopregnanolone is further suggested by studies suggesting its role in the susceptibility to developing PTSD, both in men and women [177, 178].

The role of neurotransmitters in stress and depressed states has become less clear as conceptualizations of depression have moved away from hypotheses of dysfunctional aminergic signaling toward theories of systemic dysregulation that involves and is expressed as changes in cell neurotrophic factors (e.g., BDNF), circadian physiology, immune system response, brain network function, neuroendocrine function (e.g., HPA axis), and transcriptional and epigenetic activity [179]. However, alterations in serotonergic [180], dopaminergic [181], GABAergic [182], glutamatergic [111], opioidergic [183], and noradrenergic [184] function have all been documented in depression or following stress, with their significance supported by the therapeutic effects of medications that influence these systems (SSRIs and TCAs for monoamines [185]; allopregnanolone for GABA [174]; ketamine for glutamate, and opioids [186]). This section provides examples from animal and human studies, respectively, that illustrate the myriad sex differences in signal transduction present in stress and affective disorders. Attention is given to preclinical findings of neurotransmitter function and cell-signaling differences that may underlie differences in affect regulation. In our discussion of clinical findings, we address not only male–female differences in neurotransmitter systems, but note findings related to hormonal effects as well.

### Preclinical findings

With regards to sex differences, preclinical work has demonstrated that stress can differentially affect neurotransmission in key brain regions in depression. Adult female mice have been shown, to a greater degree than males, to have increased parvalbumin mRNA expression and parvalbumin-containing cells (measures of GABAergic interneurons) in prefrontal cortex following chronic stress, molecular changes that correlate strongly with behavioral endpoints reflecting anxiety and depression [187]. In CRH-receptor deficient adult mice that display increased vulnerability to stress-related behavior, females exhibit increased sensitivity to the acute modulation of serotonin (via SSRI administration) relative to males on tail suspension, elevated plus maze, and light–dark box tests (acute stressors) [188]. This effect is hypothesized to be due to serotonergic hypofunction in prefrontal cortex as well as in hippocampus in females with CRH-receptor deficiency. (It should be noted that female hippocampal serotonin function is also decreased relative to males in wild type animals) [188]. With regards to dopamine, hyperactivity in the nucleus accumbens–ventral tegmental area (NAc-VTA) reward pathway is induced by social defeat stress in adult female, but not male, mice [189, 190], and D1-receptor activation in the NAc appears to be a uniquely important mediator of the stress-induced withdrawal phenomenon in females [189, 190]. Similarly, subchronic variable stress has been observed to increase firing in neurons projecting from the lateral habenula to the VTA in adult female, but not male, rats, a finding associated with behavioral correlates of decreased reward sensitivity [191]. In a rodent experiment of excessive glucocorticoid exposure during the late gestational period (modeling prenatal stress), dimorphic effects in adult offspring were observed on structural characteristics of dopaminergic neuronal populations and factors associated with dopamine neurotransmission, such as innervation pattern, number of receptors and transporters, as well as basal and amphetamine-stimulated dopamine release in multiple brain regions [192], again suggesting an interaction between sex, stress, and development. Ketamine, a novel antidepressant, impacts the function of glutamate and GABA receptor systems. Sex differences in ketamine's antidepressant-like effects in rodents have been explored in a number of studies [186, 193, 194], with female rodents typically demonstrating greater sensitivity to rapid and sustained antidepressant effects than males based on forced swim test immobility time, an effect mediated by estrogen and progesterone [193]. Conversely, physiologic biomarkers associated with stress-related behavioral changes were reversed following higher doses of ketamine in adult male, but not female, animals in response to chronic social isolation (another form of chronic variable stress), consistent with an increase in spine density in mPFC pre-limbic pyramidal neurons of males only [194]. How these effects relate to human depression is yet to be determined.

Other animal studies have yielded intriguing evidence for sex-differences in cellular function at individual synapses within regions implicated in depressive pathology. Some of the findings represent convergent differences (i.e., different mechanisms leading to the same functional outcome). For example, Woolley et al. showed that pre and post-synaptic hippocampal glutamate receptors are regulated by completely different estrogen receptor subtypes in male and female adult rats, with no resultant difference in glutamatergic transmission [195]. Clear examples of different functional outcomes can be seen in the studies of CRH receptor signaling in the locus coeruleus, where sex differences appear to potentiate emotional arousal in female rats [54]. As described by Bangasser and Valentino, increased coupling between the corticotropin releasing hormone (CRH) receptor and its associated G protein on the membrane surface renders female LC neurons more sensitive to CRH [54, 196]. Furthermore, cellular internalization of the CRH receptor, an adaptive process that prevents adverse effects secondary to overstimulation, occurs in males but not females in response to stress [54, 196]. While the CRH receptor is able to couple with beta-arrestin 2 (a protein involved in agonist-mediated desensitization) and be internalized in males, the G-protein outcompetes beta-arrestin 2 for binding with the receptor in females, making an internalizable complex less likely to form [196–198].

### Clinical findings

Clinical research in depression has focused primarily on sex differences in serotonin (in part due to the success of SSRIs in the treatment of mood disorders), with studies demonstrating sexual dimorphism in serotonergic function in MDD [199–202]. Positron emission tomography (PET) and single photon emission computed tomography (SPECT) studies have shown higher 5HT1A receptor concentrations, lower 5-HTT binding potentials, and decreased serotonin uptake [199, 200] in depressed adult women relative to depressed men, findings that extend across several cortical and subcortical brain regions. However, some studies have reported contrasting findings with regards to the directionality of effects (e.g., depressed men showing decreased serotonin transporter (SERT) [203]), which may reflect methodological differences, small sample sizes, or the influence of other factors affecting serotonin transmission [204]. As an example of such factors, changes in SERT in response to seasonal changes show a combined effect of both sex and genetic background, with premenopausal adult women carrying the short allele of the serotonin-transporter-linked polymorphic region (5-HTTLPR) demonstrating both higher propensity toward seasonal affective disorder and poorer ability to downregulate serotonin transporter levels during shorter photoperiods relative to men of either genotype and women with the long-allele [205]. Research on other neurotransmitter systems is fairly limited, though postmortem studies have demonstrated greater down-regulation of somatostatin, a marker of one type of GABAergic neurons, in DLPFC, anterior cingulate, and amygdala in women with MDD relative to men [206–208]. In addition, one study demonstrated increased expression of several glutamatergic genes in DLPFC in adult women with MDD compared to men [209]. From the perspective of therapeutic response, sex and hormonal status may play a role in the efficacy of monoaminergic antidepressant treatments. Some studies have reported that women respond better to SSRIs and MAOIs, and men to tricyclic antidepressants [210], though this has not been borne out by meta-analyses [210, 211]. In women, gonadal steroids appear to modify the response to neurotransmitter modulation, with premenopausal individuals showing a better response to SSRIs than postmenopausal individuals [210]. Results from one study also suggest that estrogen treatment may improve quality of life (but not depression scores) in postmenopausal women during SSRI treatment of depression [212].

### Summary

The difficulty in making generalizable statements with regards to the effect of sex on neurotransmission (e.g., being male/female produces X effects on Y neurotransmitter, resulting in Z behavioral outcome) speaks to the highly complex, dynamic, and overall poorly understood interaction between sex and neurotransmission in depressed states. However, regardless of specific effects, it is apparent that sex/sex hormones have a broad impact on the fundamental signaling processes that ultimately contribute to higher order neural phenomena implicated in psychiatric illness. Even as theories of depression centered on neurotransmitter deficits are updated, sex effects on signal transduction remain applicable to newer, more comprehensive systems-level hypotheses. For instance, it has recently been postulated that network imbalances between excitation and inhibition (E:I) may underlie several neuropsychiatric illnesses, including major depression [182, 213]. Estradiol plays a major role in this balance, as estrogen receptors can activate metabotropic glutamate (the major excitatory neurotransmitter) receptors, even in the absence of glutamate, and can increase synaptic trafficking of ionotropic AMPA receptors [214]. Estradiol also acutely regulates excitation, inhibition, and neurosecretory coupling through direct effects on calcium and potassium channel activity [215, 216]. Other neurotransmitter systems that contribute to E:I balance, as described above, are widely influenced by sex and sex hormones as well, and bear a relevant connection to behavioral outcomes. Therefore, if depression results from E:I imbalance, then that disturbance may well reflect the effects of sex on the basic signaling processes that regulate this balance.

## Transcription/translation

Animal studies have identified multiple genes and gene networks that are impacted in stress models of depression (e.g., unpredictable chronic mild stress) [26, 206, 217]. Many of these stress-related genes show marked sex-differences. For example, certain genes have been shown to be critical to behavioral dysregulation uniquely in human males and females [218], and gene networks appear to be altered in a sex-specific manner following stressful stimuli in both animals and humans [218–221]. In human studies, not only does there appear to be little overlap between the genes and gene pathways that are affected in depression in males and females, but the genes that do overlap are often regulated differently, with transcription often occurring in an opposite fashion depending on sex. In addition, studies that have attempted to correlate transcripts with physiologic function have shown that differentially expressed genes produce unique downstream effects. For example, gene associations to immune processes have been documented to a larger extent in females than males in several studies [26, 222]. Gene expression differences are, therefore, prime candidates for exploring the molecular basis of systems-level sexual dimorphism. In this section, we focus on a recent series of studies: two preclinical studies examining transcriptional profiles following stress in animals with various gene complement/sex steroid combinations; one study comparing transcriptional “signatures” in men and women with MDD, validated with a rodent model; and one study examining downstream targets of transcription in men and women with MDD.

### Preclinical findings

Sex differences in transcription of depression-associated genes could reflect hormonal effects, genetic effects, or both. Two recent studies utilized the "four core genotype" paradigm in an attempt to disentangle hormonal and genetic sources of sex differences in animals, with results suggesting a combined role of genes, acute hormonal exposure (activational effects), and developmental hormonal exposure (organizational effects) to differences in stress-induced transcription. In FCG, the testes-determining SRY gene is moved to an autosome, generating animal subjects whose gonadal sex can be made independent of their genetic sex. This yields four genotypes: XX females, XX (gonadal) males, XY males, and XY (gonadal) females. Animals may be gonadectomized and provided with hormone replacement depending on the outcome of interest, yielding several genetic complement/hormonal milieu combinations. Using FCG adult mice in a chronic variable stress paradigm, Barko and colleagues [219] demonstrated more pronounced effects of stress on gene expression for several genes responsible for dopamine and glutamate metabolism in mesocorticolimbic brain regions, as well as on gene network coordination, in female conditions (XX, gonadal female, and/or no hormone replacement) than in male conditions (XY, gonadal male, and/or testosterone-treated). In a follow-up study assessing organizational hormonal effects (FCG mice were gonadectomized but did not receive hormone replacement), both hormone exposure during critical developmental periods and genetic sex were shown to contribute to differential patterns of gene expression in mesocorticolimbic brain regions under stressed conditions [220]. The investigators also identified a set of differentially expressed “hub” genes regulated in opposite directions by stress in XY males and XX females. Of note, several of the biological pathways encoded by these differentially expressed genes were related to immune function, consonant with the putative role of inflammation in MDD.

### Clinical findings

Using RNA sequencing methodologies, LaBonte and colleagues [218] made several important observations related to sex differences in genetic expression of MDD. In addition to demonstrating little overlap in global and regional transcription between depressed adult men and women, they were able to identify distinct genetic “nodes” for critical gene networks implicated in male and female depression, a finding supported by direct genetic manipulation in mice. Across 6 brain regions implicated in depression, there was only 5 to 10% overlap between men and women of genes differentially expressed in depressed subjects, as well as little similarity in terms of the pattern of up/down regulation of genes across regions (i.e., when comparing transcription profiles from individual regions to one another). Similar findings were observed in adult mice subjected to chronic variable stress, and the significant number of differentially expressed genes shared between males of both species and females of both species suggests conservation of sexually dimorphic pathways of stress-induced pathology.

As part of the same study, the investigators utilized multi-brain region co-expression networks to evaluate transcriptional "signatures" associated with human MDD. Among the shared modules of gene connectivity (i.e., coordinate expression across brain regions) in depressed individuals vs. controls, the majority showed increased connectivity (association) in men compared to women. Their analysis identified genes *DUSP6* and *EMX1* as important nodes for gene networks implicated in MDD in women and men, respectively (*DUSP6* encoding a widely prevalent phosphatase, and *EMX1* encoding a similarly ubiquitous transcription factor), findings subsequently supported by gene knockout/overexpression in adult animals. *DUSP6* downregulation led to depressive behavior in female, but not male, mice subjected to chronic variable stress, an effect that was reversed with subsequent vector-mediated overexpression of *DUSP6*. In contrast, upregulation of the *EMX1* gene resulted in similar behavioral dysregulation in stressed males, without inducing stress susceptibility in females. An interesting finding, in line with the concept of sex-related physiologic convergence, was similar functional changes (increased excitatory postsynaptic currents in ventromedial prefrontal cortex) in both sexes as a result of genetic manipulation of either *DUSP6* (in female mice) or *EMX1* (in male mice).

A study by Seney and colleagues [223] utilized similar methodologies in a meta-analytic format to explore the differences between the male and female transcriptome in major depression in humans. Their findings replicated the minimal transcriptional overlap observed in the LaBonte study while showing a high degree of overlap in genes regulated in opposite directions in adult men and women with depression and demonstrating differences in the downstream effects of each transcriptional profile. Using gene ontology analysis, they identified genes for synapse related pathways, inner mitochondrial membrane protein complex, and G protein coupled amine receptor activity as associated with male MDD, whereas pathways related to antigen processing and mitochondrial function were associated with female MDD. In their cell-type analysis, they demonstrated that differentially expressed genes expressed in oligodendrocytes and microglia were upregulated in men with MDD but downregulated in women with MDD, while genes expressed in neurons were downregulated in men but unchanged in women. It was notable that sex differences had not been reported in the individual studies from which these data were compiled, either because sex was not considered or statistical power was not great enough in the individual studies to detect differences.

### Summary

Overall, these studies suggest significant differences between males and females in terms of genetic expression underlying stress-related pathology. Additional studies are needed to characterize the functional relevance of these differences—it will be crucial to explore how molecular differences manifest themselves on a physiologic level to produce susceptible and resilient phenotypes. Experiments such as those conducted in the LaBonte study (genetic manipulation of hub-genes identified in their network analysis) and a previous study by LaPlant and colleagues, which demonstrated both an increase in transcription of genes coding for nuclear factor kappaB (a transcription factor involved in cellular protection during stress) following ovariectomy and an association of increased nuclear factor kappaB with susceptibility to stress [221], are likely to provide valuable links between gene expression, sex, and dysregulated behavior.

## Epigenesis

Epigenetic mechanisms (e.g., DNA methylation; histone methylation and acetylation) serve to alter gene expression through modification of nucleosomes (DNA and histone proteins) without changing the fundamental nucleotide sequence. Methyl groups covalently linked to DNA at specific cytosine–phosphate–guanine sites (CpGs), result primarily (albeit not exclusively) in gene repression [224]. Histone acetylation serves as an opposing process, with acetyl groups added to the N-termini of histone proteins to ultimately remodel chromatin and allow for enhanced/increased DNA transcription [225]. Other epigenetic modifications include ubiquitination, phosphorylation, sumoylation, and ribosylation, as well as post-transcriptional modifications, such as those induced by microRNA and sRNA. Epigenetic sex differences have been documented across species and in multiple tissues, including blood [226, 227], placenta [228], liver [229, 230], pancreas [231], muscle [232], heart [233], and brain [234–239]. Epigenesis appears to be a critical mechanism by which sexual differentiation occurs during the neonatal period and puberty [240–242]. Evidence is emerging for sex differences in epigenesis for several disease conditions as well, including diabetes [243], autoimmune diseases [244], cardiovascular disease [245], and cancer [246]. Methylation and acetylation have been shown to underlie behavioral adaptations to chronic stress in animal models [247–249], and convergent associations between depression and epigenetic modifications have been demonstrated in human clinical studies [250], highlighting the role of these processes in dynamic emotional states. Given its reversible nature and plausible link to episodic (as opposed to continuous or progressive) dysfunction, epigenesis represents an appealing hypothesis for regulation and dysregulation of mood and behavioral states [251–253]. We review some of the recent clinical and preclinical evidence for epigenetic sex differences below. Particularly notable is the association between sex-specific transcriptional profiles and both DNA methylation and micro RNA networks, suggesting these processes work together as part of a coordinated response to stress. Clinical findings are limited, and we present the example of epigenetic changes in human offspring secondary to maternal behavioral characteristics during pregnancy.

### Preclinical findings

Though the research is still in its nascent stages, evidence from preclinical studies supports the notion of sex-specific epigenetic adaptations to stress. One study looking at the effects of chronic variable mild stress on CRH gene methylation and epigenetic enzymes (DNA methyltransferases, histone acetyltransferases) demonstrated overall lower methylation in adult female rats following stress across several CRF-containing brain areas, though with pronounced regional effects [254]. In the paraventricular nucleus (PVN), total DNA methylation of the CRH gene was consistently higher in stressed females (consistent with decreased expression) relative to female controls, an effect not seen in males. Conversely, in the bed nucleus of the stria terminalis (BNST), CRH methylation was decreased in stressed males relative to controls, with females showing no effect. In amygdala, stress resulted in decreased total methylation for females relative to males. Following stress, CREB-binding protein, a histone acetyltransferase, was increased in female BNST, and mRNA for histone deacetylase-5 was decreased in male amygdala. All of these differences were reflected by sex-specific modifications to one or more specific CpGs, as well as differences in expression of c-Fos, FosB, CRH mRNA, and CRH peptide.

DNA methylation appears to mediate expression of sex-specific transcriptional profiles associated with susceptibility to stress-induced behavioral changes. A study by Hodes et al. [255] examined transcriptional regulation in nucleus accumbens of adult mice in response to a chronic stress paradigm. These authors demonstrated that conditional deletion of DNA methyltransferase 3a (Dmnt3a) resulted in increased behavioral resilience in female mice, defined as resisting changes in behaviors normally produced by stress, including decreased grooming time, increased latency to eating in the novelty suppressed feeding paradigm, decreased sucrose preference, and reduced active coping in the forced swim test. This effect was not noted in males, as males without the knockout were already behaviorally resilient. Using RNA sequencing, they demonstrated that Dnmt3a knockout resulted in alterations of the stress-associated transcriptional profile, creating a hybrid of male and female phenotypes associated with increased resilience in female animals. This suggests that Dnmt3a may be a more important modulator of stress susceptibility in females than in males, who appear to possess mechanisms counteracting its deleterious effects. Notably, investigators also found Dnmt3a to be increased in postmortem samples of both male and female humans diagnosed with MDD [255].

MicroRNA regulation is another epigenetic process being explored in terms of its relationship to affective disorders. MicroRNAs are small RNAs involved in the post-transcriptional regulation of mRNA, acting via base-paring with mRNA to cause cleavage, destabilization, and decreased translation. This mechanism is ubiquitous and evolutionarily conserved, as well as widely present in the central nervous system [256, 257]. Research has shown that the neonatal microRNA environment in the hypothalamus is both sexually dimorphic and dynamically responsive to estrogen, suggesting that this additional layer of gene regulation is crucial to sexual differentiation and fetal epigenetic programming [258]. Pfau and colleagues found evidence to suggest that adult mouse microRNA networks are regulated in a sex-specific way in response to stress, and that these effects are part of a larger, coordinated response involving transcriptional and post-transcriptional regulation that is unique to each sex [222]. Using genome wide analysis of sex-specific microRNA and mRNA transcriptional profiles, they demonstrated that, analogous to transcriptional profiles for other stress-related genes, microRNA transcriptional profiles induced by stress were largely non-overlapping between males and females. In addition, similar to transcriptional findings, these miRNA profiles demonstrated markedly different associations to molecular pathways and functions in each sex. Overall, males, but not females, showed a robust transcriptional and post-transcriptional response to stress, suggesting a form of “active” resistance leading to behavioral resilience [222]. Male miRNA functional pathways overlapped to a greater degree with the pathways of other differentially expressed genes involved in the stress response than did female miRNA pathways, pointing to a potentially greater role of miRNA in stress-responsive molecular processes in males. (However, because the enrichment analyses were lower powered in females due to smaller gene lists, this effect may have been exaggerated). As there was minimal overlap in genes, miRNAs, and functional processes related to the stress response between males and females, these results also support the notion that the female response to stress is unique, and not simply an attenuated version of the male response.

### Clinical findings

Clinically, there has been a recent focus on ways in which prenatal experience can affect subsequent susceptibility to mental illness [259–261], with female offspring being more susceptible to affective dysregulation and males more likely to suffer from memory and learning impairment if exposed to prenatal stress [262]. Early DNA methylation appears to play a role in this vulnerability, with evidence suggesting that predisposition to affective disorders in children is associated with sex-specific methylation of critical HPA-axis genes. In a recent experiment [263], it was hypothesized that anxious-depressive behavior in young female, but not male, children would be accompanied by greater methylation of the NR3C1 gene, a glucocorticoid receptor gene implicated in HPA feedback mechanisms [264]. In a prior study, mood worsening in mothers following delivery (i.e., low prenatal depression followed by high postnatal depression) was associated with methylation of NR3C1 in their offspring, an effect that was reversed by early postnatal maternal stroking of the infant [265]. In addition to evidence supporting the authors' behavioral hypothesis that girls were more likely to experience depressive symptoms in the setting of mismatched maternal prenatal–postnatal depression (in this case, either low prenatal depression followed by high postnatal depression, or vice versa), the results of their follow-up study showed that prenatal–postnatal mismatch had strong effects on NR3C1 methylation in girls only, with low prenatal depression followed by high postnatal depression resulting in the largest increase [263]. Higher NR3C1 methylation predicted anxious-depressive behavior at 14 months in girls, whereas no association was seen for boys. Whether this association persists into adolescence and adulthood has not yet been determined.

### Summary

Epigenetics represents a promising area of research for affective disorders. The relationship between the epigenetic response and the transcriptional response to stress suggest these processes act in a coordinated fashion to elicit broader physiologic effects. Additional clinical research is needed. Current findings are concordant with the notion that early epigenetic changes play a role in subsequent behavioral vulnerability.

## Discussion

Taken as a whole, these studies provide considerable evidence for sex differences in the CNS structures and processes that contribute to affective regulation. Sex and sex steroids exert specific but wide-ranging effects on the brain (in general) and affective regulation (in particular) at virtually any level of investigation, from molecular to systems level, from synapse structure to network regulation (see Table 1). Because mood dysregulation does not reside in a specific brain region nor does it rely solely on changes at any one physiologic level, cause-and-effect with regards to these differences cannot be inferred. A change in transcription, for instance, may be compensated for by other changes resulting in no difference in the ultimate outcome measure. As such, sex differences can exist without behavioral consequence. Nonetheless, we can conclude the following: (1) sex and sex steroids create a context that determines or influences the structures and processes underlying behavior, including affective regulation and response; (2) if we are attempting to understand the physiology of behavior by studying only one sex, we are likely to fail to uncover alternate molecular pathways that would help define the most critical loci at which physiologic adaptation to stressful stimuli fails to occur; and (3) our efforts to develop new therapeutics may be advanced by exploiting the observation that manipulation of reproductive steroids can regulate mood in susceptible subgroups of women; i.e., sex steroids can serve as probes for defining the changes in cell signaling that precede and accompany changes in affective state.

**Table 1:** Examples of sex differences and sex hormone effects, by level of observation

Level of observation	Data source	Basal sex difference	Sex difference in stress and affective disorders	Sex hormone effect (non-stress)	Sex hormone effect (stress and affective disorders)
Brain structure	Animal	Sexually dimorphic brain regions, (e.g., mePOA) [41, 268]; locus coeruleus structural dimorphism [54]	Regional morphology differences following prenatal stress [55–60]	E2 impact on physiologic development [269]	E2 neuroprotective in brain injury [270]; neuronal loss in the prefrontal cortex, hippocampus, hypothalamus, and amygdala following OVX [271]
	Human	Women increased gray/white matter ratio [42, 43]; gray matter density differences in several brain regions [45]; different developmental rates[46, 47]; cortical surface area trajectory [272]; volumetric differences at birth [273]	In childhood stress: lower gray matter thickness and caudate volumes in females, decreased thickness of rostral anterior cingulate cortex in males [64]; amygdala differences following prenatal stress [65–69]; differences in cortical gyrification [70]	Volumetric changes during different menstrual phases [274, 275]; regional differences between OCP users and cycling women [275]	Effects of menstrual cycle on hippocampus in PMDD [276]
Network connectivity	Animal	Sex differences in circuits implicated in parenting behavior [277]	Differential network activation in response to pain [278–280]; differences in network organization following prenatal ethanol exposure [281]	Dendritic spine density fluctuation during estrous cycle [166]; E2-dependent reward circuitry [282]	Hippocampal/PFC remodeling following stress mediated by E2 [122]
	Human	DMN [283]; white matter [83–85]	Weakening of the iFC of the DMN in female adolescents, predicting greater internalizing symptoms [127]	Reward [96]	Network response to hormonal manipulation [131, 138]
Signal transduction	Animal	Neurotransmission, many isolated differences [16]	PFC GABA function/reward [187]; higher HPA activity following stress in females [16, 284]	Neurotransmission [285]; reward processing [169, 170]	E2 effects on neurotransmission/cell signaling/feedback [142, 286–288]; Testosterone effects on HPA response to stress [22]
	Human	GABA [289]	5HT [199, 200], GABA [207, 208], glutamate [209]	E2 x genetic background influence on dopamine-mediated reward [171]	Hormone withdrawal/allopregnanolone in PPD [174]; Altered estradiol-dependent cellular Ca 2 + homeostasis and endoplasmic reticulum stress response in PMDD [290]
Transcription/Translation	Animal	Basal differences secondary to direct sex hormone effects [291]	Differential transcription—minimal overlap in stress-associated genes [218–220]	Direct sex hormone effects (e.g., classical sex hormone effects) [292]	change in protein expression associated with depressive behavior following OVX [271]
	Human	Basal differences secondary to direct sex hormone effects [291]	transcriptional differences by sex in MDD and controls [218, 223]	Direct sex hormone effects [291]	Differential transcriptional effect of E2 and P4 in PMDD vs. controls [293]
Epigenesis	Animal	Widespread basal differences, including brain [233, 234, 239]	DNA methyltransferase [254], miRNA differences [222]	E2/estrogen receptors regulate DNA methylation, demethylation, histone modification, chromatin remodeling [294]	In-utero stress produces differential epigenetic response in offspring [295, 296]
	Human	Widespread basal differences, including brain [227–232]	Methylation differences following prenatal stress [263]	E2 effect on epigenesis of puberty [240, 241, 297]	Differential expression of ESC/E(Z) complex (a gene silencing complex that functions via methylation) by E2 and P4 in PMDD vs. controls [293]

Examples of sex differences and sex hormone effects at each organizational level. This table serves as a scaled-down version of our framework presented above. As in the body text, the content presented in each box is meant to provide illustrative examples within each category rather than a comprehensive list of findings. Examples for both basal/non-stressed conditions and stressed/affective disorder conditions are shown, and are separated according to human and animal research. *mePOA* medial preoptic area, *E2* estradiol, *P4* progesterone, *OCP* oral contraceptive, *PPD* peripartum depression, *PMDD* premenstrual dysphoric disorder, *MDD* major depressive disorder, *PFC* prefrontal cortex, *iFC* intrinsic functional connectivity, *DMN* default mode network, *HPA* hypothalamic pituitary axis, *5HT* serotonin, *OVX* ovariectomy

Unfortunately, sex as a moderating variable has often been ignored or intentionally excluded from research studies due to concerns about added complexity (e.g., studies would need to control for factors, such as menstrual/estrous cycles) and sample size (greater numbers are needed to power studies looking at sex differences a priori). Combined with our lack of understanding about the etiology of depression in general, this has left us with little evidence for generalizable sex-dependent characteristics associated with "male" or" female" depression. As neuroscientific and genetic techniques yield a better understanding of affective physiology, and as newer, more efficacious therapeutics with unique mechanisms of action such as ketamine and neurosteroids [174, 266] become better studied, a clearer picture may emerge regarding how sex ultimately influences susceptibility, symptom expression, and treatment.

Additional considerations for future work include the following: addressing, for any sexual dimorphism, whether findings reflect organizational, activational, or genetic differences; exploring other contextual factors that interact with stress/sex differences, including point in lifespan, past experience, and the different environments (both internal and external) to which males and females are subjected; and studying the effects of different stressors, particularly in human brains that are obviously more complex than the rodent models from which much of the evidence is derived. As an aspiration, relevant sex-specific targets may lead to the development of more precise therapeutics, with requisite consideration of safety/risk, practicality, and effectiveness. The extant research makes it clear that interventions in one sex cannot be assumed to have equivalent effects in the other, requiring studies powered to detect effects in both males and females. For instance, a treatment such as allopregnanolone that has known efficacy and safety profiles in women must be rigorously studied in men if it is to be considered for use in this population.

**Fig. 1:**
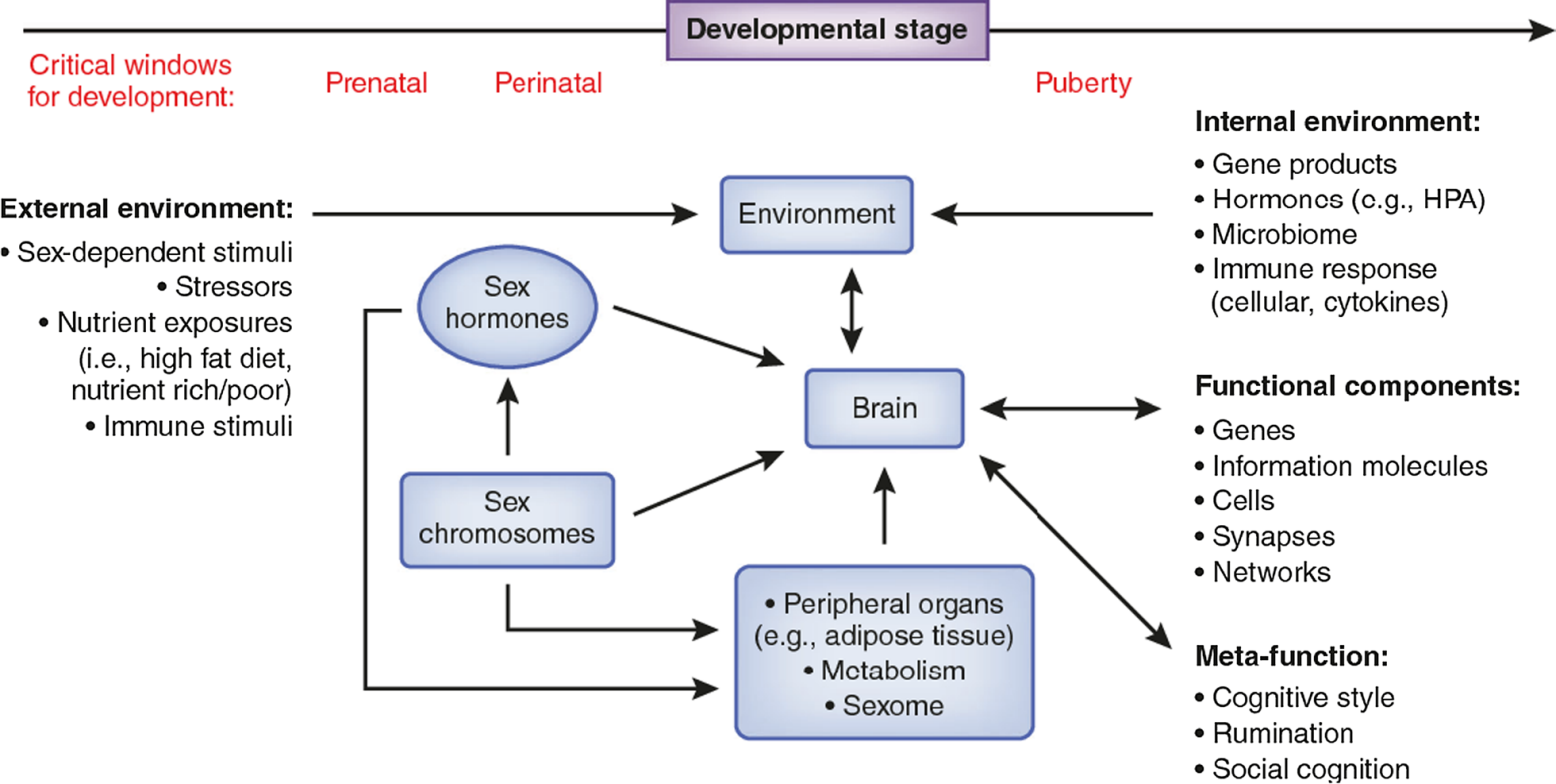
Depiction of the levels at which sex influences brain function. Sex modulates brain function and behavior through both acute effects (i.e., activational effects), as well as the programming of brain sensitivities during critical periods (i.e., organizational effects). These levels interact with one another in a dynamic fashion and include components within the central nervous system, in peripheral body systems, and external to the organism altogether (e.g., external environmental effects). Though not specifically explored in this paper, non-CNS physiologic factors including microbiome effects, immune response, and differences in peripheral organ/metabolic function are important when considering how sex influences the brain. Non-physiologic factors, such as social responses from others and meta-cognitive function, also play a significant role (from [38])

Sex and sex hormone signaling occupy a central role in the formation, programming, and functional orchestration of the brain. As such, attempting to define the specific effects of sex on the already mysterious and complex processes giving rise to affective disorders is daunting. Despite our inability to define the role of sex signaling factors in depression, their potential impact can be seen throughout the brain. Translation of described sex differences in stress-related disorders into actionable data will require a far more comprehensive picture of the link between predisposing factors and behavioral outcomes, ideally in the form of novel, predictive, biosignatures. Though not specifically focusing on sex, Hultman and colleagues [267] used machine learning to distinguish profiles of depression-like behavior from profiles of the susceptibility to depression-like behavior. With EEG, these investigators provided evidence of a network-level, spatiotemporal, dynamic signature associated with vulnerability to depression that *preceded* stress, and that a) was distinct from the dynamic signature associated with behavioral dysfunction following the stressor; b) differentiated susceptible mice from resilient mice; c) was present in three independent models of MDD; and d) was not affected by antidepressant manipulations. This type of mesoscopic phenotype may serve as an outcome measure that integrates sex differences at multiple levels and across developmental timepoints, facilitating the assessment of how sex differences at the genetic or cellular level influence brain dynamics associated with the vulnerability to or experience of affective dysregulation. Nonetheless, despite the impressively large number of ways in which sex may plausibly influence the development and expression of affective disorders (see Fig. 1), we must look to the future to transform the current state of isolated findings into a more coherent picture of how sex differences meaningfully impact the regulation and dysregulation of affect.

## Perspectives and significance

Abundant evidence exists for biological sex differences that may contribute to both susceptibility to depression and sex-differences in its prevalence. Nonetheless, our lack of understanding of the ontogeny of depression itself precludes determination of the etiopathogenic significance of reported sex differences. To address the more general question, “Why would you think that sex would influence depression,” we present examples of the role of sex in regulating neurobiology at five related levels of observation. We believe that this framework for organizing observations from the literature may facilitate a less particularized, more integrative approach to examining the role of sex in depression and, by so doing, also generate a more comprehensive picture about the relationship between predisposing factors and behavioral outcomes in affective illness.

## Data Availability

Data sharing is not applicable to this article as no data sets were generated or analyzed during the current study.
